# Uptake of a web-based oncology protocol system: how do cancer clinicians use eviQ cancer treatments online?

**DOI:** 10.1186/1471-2407-13-112

**Published:** 2013-03-12

**Authors:** Julia M Langton, Nicole Pesa, Shelley Rushton, Robyn L Ward, Sallie-Anne Pearson

**Affiliations:** 1 Faculty of Pharmacy, University of Sydney, Sydney, NSW, Australia; 2 Cancer Institute New South Wales, Sydney, NSW, Australia; 3 Prince of Wales Clinical School, University of New South Wales, Sydney, NSW, Australia

**Keywords:** Clinical decision support systems, Evidence-based practice, Cancer chemotherapy protocols, Clinical oncology, Health personnel

## Abstract

**Background:**

The use of computerized systems to support evidence-based practice is commonplace in contemporary medicine. Despite the prolific use of electronic support systems there has been relatively little research on the uptake of web-based systems in the oncology setting. Our objective was to examine the uptake of a web-based oncology protocol system (http://www.eviq.org.au) by Australian cancer clinicians.

**Methods:**

We used web-logfiles and Google Analytics to examine the characteristics of eviQ registrants from October 2009-December 2011 and patterns of use by cancer clinicians during a typical month.

**Results:**

As of December 2011, there were 16,037 registrants; 85% of whom were Australian health care professionals. During a typical month 87% of webhits occurred in standard clinical hours (08:00 to 18:00 weekdays). Raw webhits were proportional to the size of clinician groups: nurses (47% of Australian registrants), followed by doctors (20%), and pharmacists (14%). However, pharmacists had up to three times the webhit rate of other clinical groups. Clinicians spent five times longer viewing chemotherapy protocol pages than other content and the protocols viewed reflect the most common cancers: lung, breast and colorectal.

**Conclusions:**

Our results demonstrate eviQ is used by a range of health professionals involved in cancer treatment at the point-of-care. Continued monitoring of electronic decision support systems is vital to understanding how they are used in clinical practice and their impact on processes of care and patient outcomes.

## Background

The medical evidence-base is increasing exponentially, making it virtually impossible for individual healthcare professionals to maintain an up-to-date and comprehensive knowledge of their field of practice [1-4]. In an attempt to address this problem, professional bodies and healthcare organizations have invested in the development of evidence-based resources that are available electronically. Like many other specialties, oncology practice has taken advantage of the growing use of web-based technology by developing online guideline and protocol systems. These have been developed across multiple jurisdictions but originate mostly from North America and Europe [5].

Electronic decision support has the capacity to improve the processes of care and patient outcomes in oncology practice. But the provision of computer support does not guarantee uptake, and utilization of systems at the point-of-care is crucial if systems are to improve the quality of clinical practice [6]. Despite the growing number of web-based oncology systems around the globe, there has been limited evaluation of the use and impact of electronic support systems in the cancer treatment setting. However, there is some evidence demonstrating electronic oncology systems are adopted readily by clinicians and are seen as integral in cancer treatment delivery [7]. Moreover, they have been shown to increase protocol compliance and reduce chemotherapy prescribing errors and adverse events [8-12].

In this article we focus specifically on an Australian web-based protocol system, eviQ (previously known as CI-SCaT). eviQ is a web-based repository of nearly 1,300 peer-reviewed cancer treatment protocols managed under the auspices of the Cancer Institute New South Wales (NSW) [https://www.eviq.org.au/]. The system underwent a major rebuild and rebranding in 2009 to better meet the specific needs of oncologists, nurses, primary health care physicians, pharmacists, and patients. Evaluations of the system to date have been primarily qualitative, with a focus on barriers to use at the point-of-care [11,12]. However, the new platform increases opportunities to conduct longitudinal quantitative research based on web-logfile analysis. As such, the aim of this study is to describe the patterns of eviQ use by clinicians practising in the Australian healthcare setting.

## Methods

### Study design

This is a study of web-logfiles generated from eviQ over a 26-month period. The study period coincides with the launch of the new platform in October 2009 and covers the first two years of operation until December 2011. The previous platform (CI-SCaT) was taken offline on March 31 2010 to allow sufficient opportunity for users to transition and register on the eviQ website.

### Study setting

In Australia, oncology protocols are delivered primarily in the ambulatory care (outpatient) setting at metropolitan hospitals (university-affiliated, tertiary referral centers covering geographic areas of around 75 square kilometers), regional centers (with catchments up to 1,200 square kilometers) and rural hospitals (with catchments up to 3,400 square kilometers).

eviQ is managed by the Cancer Institute NSW, a government funded agency established to improve cancer control in Australia’s largest state, NSW. eviQ primarily targets health professionals involved in implementing cancer care by providing detailed and extensive instructions on how to deliver evidence-based treatments safely and appropriately. Treatment information encompasses adolescent and young adult care, cancer genetics, haematology, haemapoietic progenitor cell transplants, medical oncology, nursing, primary health, palliative care and radiation oncology. The site comprises over 1,300 protocols, developed by a consensus process involving specialist physicians, nurses, pharmacists and allied health practitioners from across Australia. Each protocol undergoes a comprehensive review every 1 to 2 years. While the primary eviQ target audience is health professionals, the site also publishes information tailored specifically to cancer patients and their carers.

### Data sources and analysis

For the purposes of this evaluation we used two data sources, both of which have different capabilities in terms of understanding eviQ use. Outputs from both data sources were converted to Microsoft excel 2010 format for analysis. We report demographic variables for all registrants at the end of our study period (December 31 2011) and patterns of use during a typical month based on eviQ logfiles. For the latter, we examined rates of use across the last three months of the study period (October through December, 2011). Rates of overall use were lowest in December (1,145 hits/100 registrants) most probably due to the holiday period, but were comparable in October (1,499 hits/100 registrants) and November (1,418 hits/100 registrants). We selected October 2011 to represent a ‘typical’ month and all subsequent analyses focused exclusively on this period.

### eviQ platform

The eviQ secretariat provided the research team with access to de-identified data from the eviQ platform. Demographic registrant and logfile data were obtained on-site at the Cancer Institute NSW in unit record format (stripped of personal identifiers such as usernames). The eviQ platform has the capacity to generate data on the characteristics of all registered users including registrant type (individual clinician or unit registration), health setting (primary care or hospital), health sector (public, private, or both), geographical location of practice, clinician group, years of oncology experience, and source of referral to the eviQ website. This information is reported by users upon registration and website registrants are prompted to update this information on an annual basis. Further, logfiles also monitor webhits, defined as one click anywhere on the eviQ website, that can be stratified by any of the aforementioned variables (e.g., clinician type, years of oncology experience) and the time at which the webhits occur (e.g., time of day, month, year). However, the current eviQ logfile reports are aggregated and do not have the capacity to determine the content accessed according to health professional groups.

As such, we report on the following:

Registrant characteristics

We report the characteristics of individuals and units identifying themselves as Australian health professionals upon site registration. We report the number of new health professional registrations by month for the period October 2009 to December 2011 and the demographic characteristics of all registrants at the end of the study period.

Patterns of eviQ use

We examined webhits during standard clinic hours (Monday to Friday between 08:00–18:00) compared with use outside clinic hours. This approach has been used previously in logfile analyses as a proxy for point-of-care use in Australian clinical practice [13]. Moreover, the majority of chemotherapy and radiotherapy cancer treatments are delivered during these times.

We stratified our analysis by registrant type (individual or unit registration), individual clinician group (medical, nursing, pharmacy and radiation therapists) and years of oncology experience. As is standard in logfile analysis, we report both the volume of webhits and hits/100 website registrants [14].

### Google analytics

The eviQ secretariat provided the research team with access to their Google Analytics profile from which we extracted data of interest. Google Analytics’ reports provide data on website traffic and allows for the examination of the intensity of eviQ use for all registrants including number of visits and unique visits to eviQ. A visit is defined as a registrant logging on to eviQ for up to four hours, unless the registrant terminates their visit by logging off or leaving the website. Google Analytics produces aggregated data relating to typical user sessions including number of pages accessed and time spent on eviQ during a typical visit for a defined period (e.g., visits in a typical week or month). Google Analytics also has the capacity to provide data on content accessed and time spent on specific eviQ pages (e.g., time spent on a particular chemotherapy protocol page).

Data from Google Analytics does not distinguish between different user groups (e.g., health professionals’ vs. consumers), limiting the capacity to undertake analyses on how different groups access eviQ. However, given that health professionals comprise 92% (n = 14,800) of eviQ registrants, Google Analytics output will be heavily influenced by patterns of use by health professionals.

Using this data source, we report on the following during a typical month (October 2011):

Typical user sessions

We report the total number of eviQ visits, average number of pages/visit, average visit duration, and mode of access (mobile device or computer). Google Analytics output is averaged across all users such that we cannot calculate descriptive statistics such as median and range for these data.

Content accessed

We report the top 100 pages accessed and time spent on these pages. The top 100 pages were grouped according to their content into the following categories: login/registration; transition pages (such as tabs directing users to specific content); and cancer treatment content pages (chemotherapy protocols or supportive treatment information). We used the time spent on each of the 100 pages to calculate the range and median time spent on pages according to the abovementioned categories. To better understand the range of clinical content accessed by eviQ users, we also conducted an analysis of the top 100 cancer treatment content pages (excluding all login, registration, and transition pages). We present total, median and range of page views according to the following categories: medical oncology, haematology, radiation oncology, and supportive treatment information.

### Ethics

Ethics approval to monitor eviQ utilization using eviQ web-logfiles and Google Analytics was obtained from the NSW Population and Health Services Research Ethics committee (approval number HREC/10/CIPHS/70).

## Results

### Registrant characteristics

At December 31, 2011 there were 16,037 eviQ registrants, the majority of whom identified themselves as Australian health professionals (85.5%, n = 13,711), followed by consumers (7.7%, n = 1,237), and health professionals practising outside Australia (6.7%, n = 1,089).

**Figure 1:**
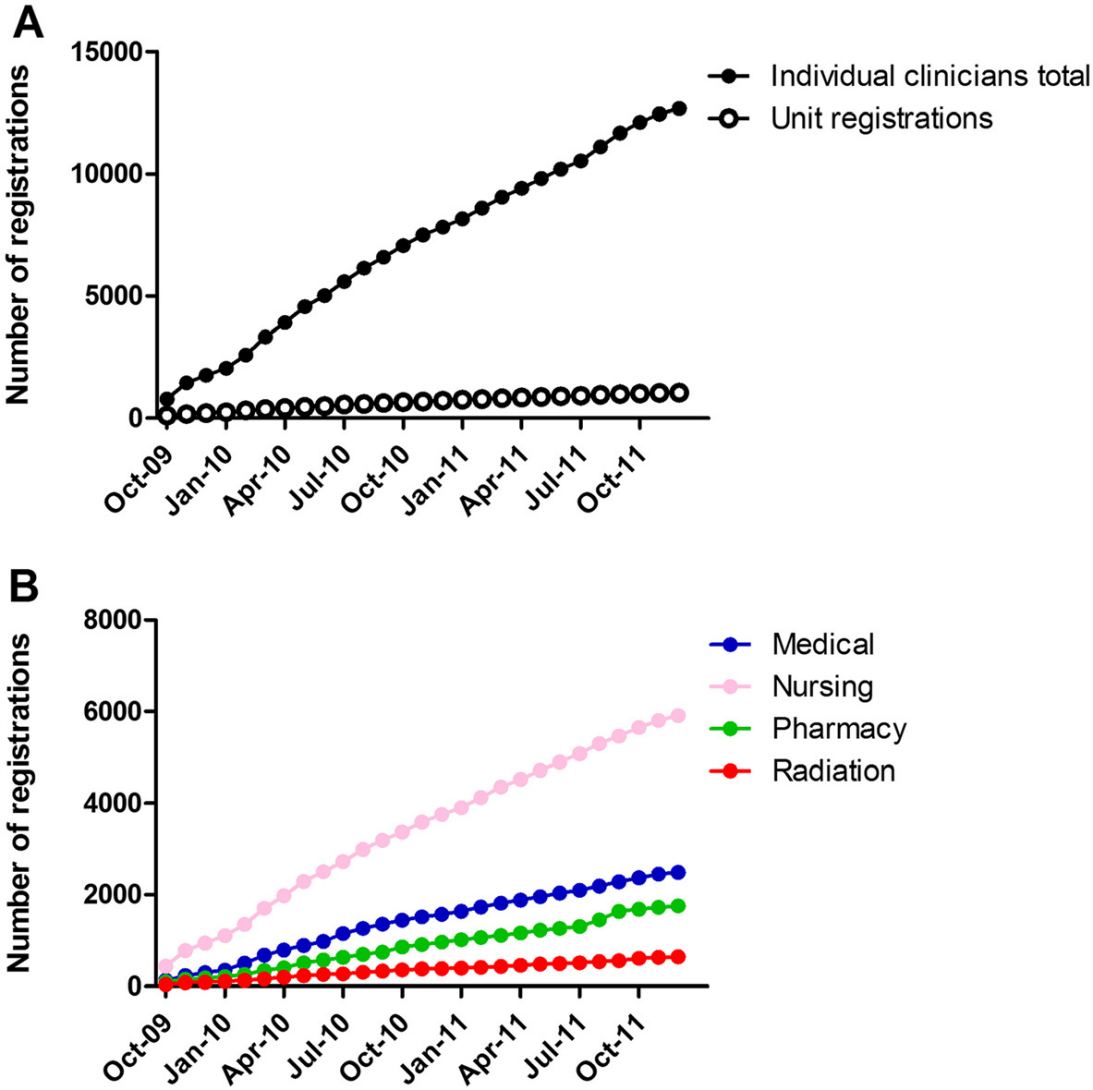
**Cumulative eviQ registrations from October 2009 through December 2011. A**) Number of registrations for all individual clinicians and units; **B**) Number of registrations for the four largest groups of individual clinicians: medical, nursing, pharmacy, and radiation therapy.

Australian health professionals were registered as individuals (92.4%, n = 12,688) or units (7.6%, n = 1,043) [Table 1]. Registrations for individual clinicians and units rose steadily over the study period with a median of 480 (range 237–889) new registrations each month (Figure 1).

**Table 1:** Characteristics of eviQ Australian health professional registrants at the end of the observation period (December, 2011)

		**Unit registration 1,043(7.6)**	**Individual clinician 12,668(92.4)**
		*n(%)*	*n(%)*
**Health setting**	*Primary health*	212(20.3)	3,009(23.8)
	*Hospital*	831(79.7)	9,659(76.3)
**Health sector**	*Public*	724(69.4)	9,005(71.1)
	*Private*	259(24.8)	2,259(17.8)
	*Both*	33(3.2)	632(5.0)
	*Not specified*	27(2.6)	772(6.1)
**Location**	*New South Wales*	378(36.2)	5,065(40.0)
	*Victoria*	236(22.6)	2,577(20.3)
	*Tasmania*	41(3.9)	405(3.2)
	*Australian Capital Territory*	12(1.2)	280(2.2)
	*Queensland*	212(20.3)	2,427(19.2)
	*South Australia*	79(7.6)	856(6.8)
	*Western Australia*	73(7.0)	873(6.9)
	*Northern Territory*	12(1.2)	181(1.4)
**Clinican group**	*Medical*	-	2,491(19.7)
	*Nursing*	-	5,909(46.7)
	*Pharmacy*	-	1,755(13.9)
	*Radiation*	-	640(5.1)
	*Other*^*a*^	-	1,873(14.8)
**Professional experience**	*< 5 years*	-	6,348(50.1)
	*5 – 10 years*	-	1,608(12.7)
	*> 10 years*	-	2,756(21.8)
	*Not specified*	-	1,956(15.4)
**Source of referral to eviQ**	*Peer/colleague*	-	7,899(62.4)
	*eviQ Education session*	-	877(6.9)
	*Conference/Booth*	-	409(3.2)
	*Internet search*	-	583(4.6)
	*Cancer Institute communication*	-	1,401(11.1)
	*Professional organisation*	-	985(7.8)
	*Other*	-	481(3.8)
	*Not specified*	-	33(0.3)

^a^Other: Academic, Allied Health, Clinical Information Manager, Medical Physicist, ‘Other- not specified’.

The majority of individual clinician registrants nominated they were practising in the public hospital setting (71.1%) and this is where most unit registrations were also located (69.4%). Most of the individual and unit registrations originated from the state of NSW (40.0% and 36.2% respectively) [Table 1].

The largest individual clinician group was nursing (46.7%) followed by medical doctors (19.7%), pharmacists (13.9%), and radiation therapists (5.1%). Approximately half of individual clinicians had less than five years’ oncology experience (31.2% had less than 2 years and 18.9% had 2–5 years’ experience). The most common eviQ referral sources were colleagues (62.4%), followed by Cancer Institute NSW communications (11.1%). Unit registrations are likely to represent a group of health professionals with varying years of clinical experience. As such, we did not report further on these registrations.

### Patterns of eviQ use

**Figure 2:**
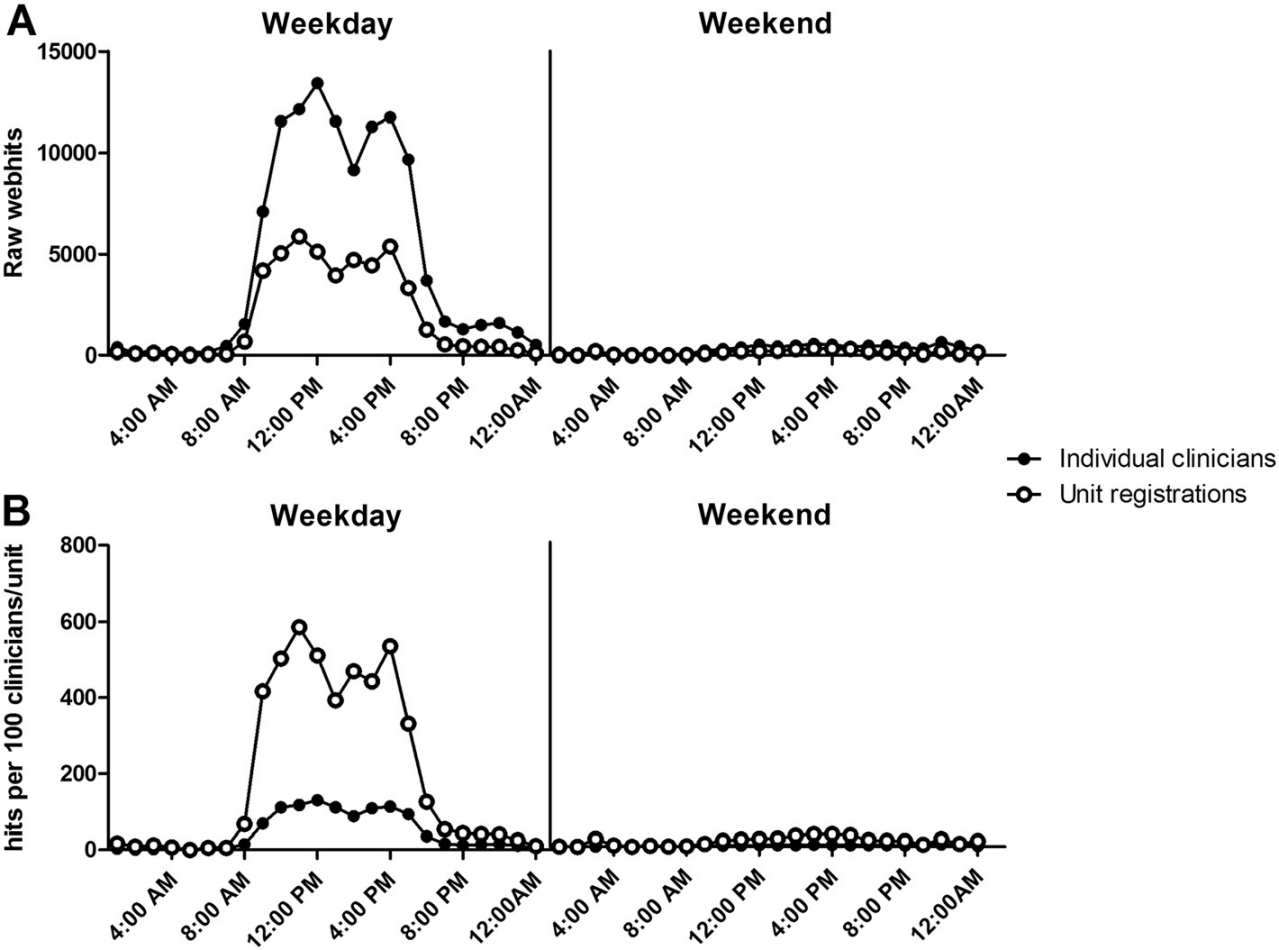
**eviQ webhits by time of day for individual clinicians and unit registrations in October 2011. A**) Raw webhits; **B**) Rates of use: hits per 100 eviQ registrants (clinician or unit). Individual clinicians include medical, nursing, pharmacy, and radiation therapy.

**Figure 3:**
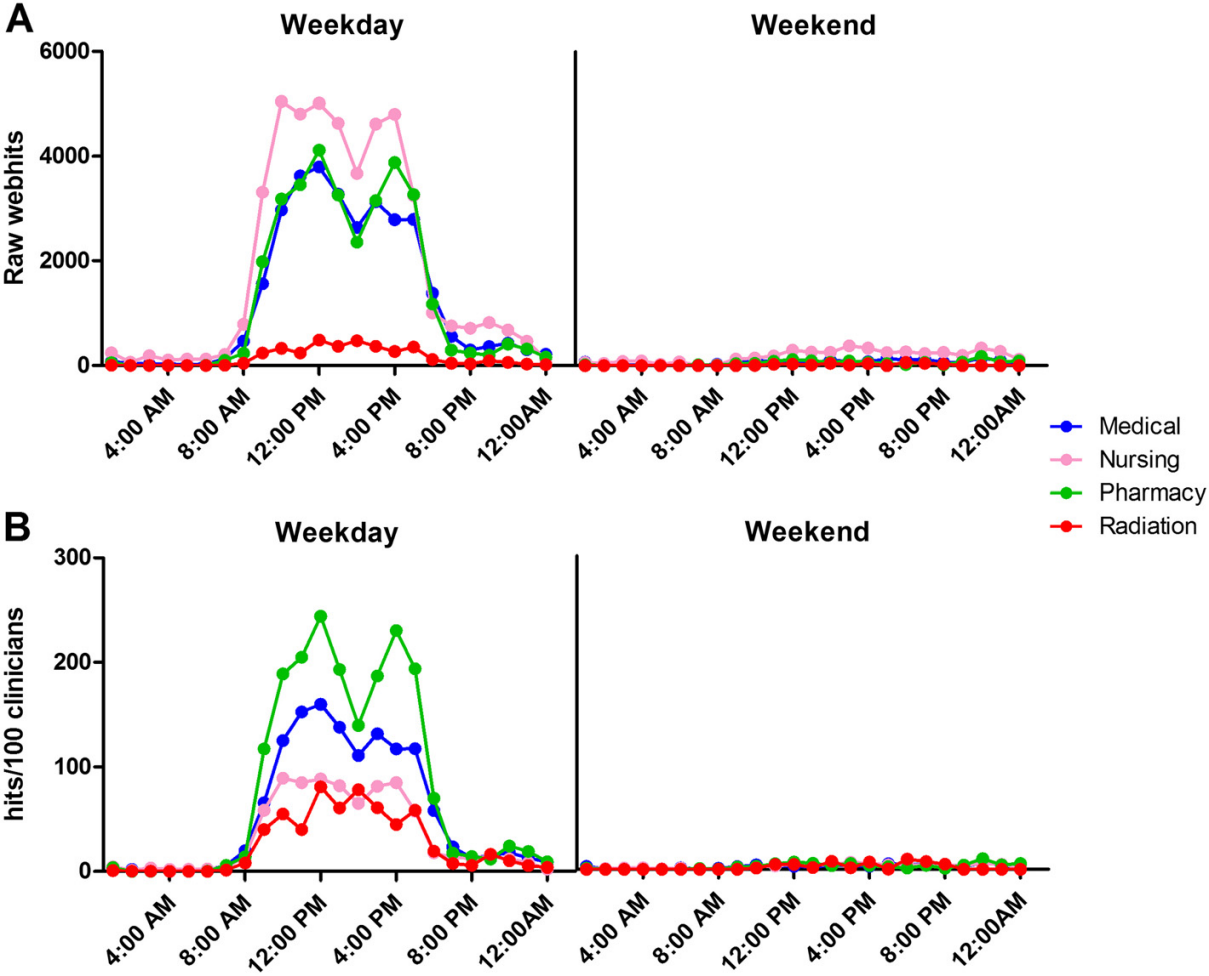
**eviQ webhits by time of day for the largest individual clinician groups in October 2011. A**) Raw webhits; **B**) Rates of use: hits per 100 individual clinicians. Individual clinicians include medical, nursing, pharmacy, and radiation therapy.

In a typical month (October 2011) there were a total of 169,647 webhits, 86.5% of which occurred during standard clinic hours (08:00–18:00; Monday to Friday). The volume of webhits was generally proportional to the number of registrations, with the largest registrant groups having the largest number of hits [Figures 2 and 3].

Not surprisingly, units had more than three times the webhit rate of individual clinicians (4,997 hits/100 versus 1,159 hits/100 site registrations) [Figure 2]. Further, pharmacists had at least 1.4 and up to three times the webhit rate (1,962 hits/100 site registrations) of other clinical groups such as medical doctors, nurses, and radiation therapists (1,373, 885 and 653 hits/100 site registrations, respectively) [Figure 3].

We also found that irrespective of professional group, clinicians with 5–10 years’ experience had 1.5 times the webhit rate of clinicians with less than 5 years’ experience (1,817 versus 1,184 hits/100 site registrations) and 1.4 times that of clinicians with more than 10 years of experience (1,817 versus 1,284 hits/100 site registrations).

#### Typical user sessions

In October 2011, there were 20,611 eviQ site visits, 7,458 of which were by unique visitors. A typical visit was 7 minutes 33 seconds in duration and 9 pages were viewed. Most visits were accessed from a computer (99%, n = 20,326), with the remainder via a mobile device (1%, n = 285).

#### Content accessed

In October 2011, there were 184,812 eviQ page views. This figure, based on Google Analytics page views, reflects data from all registrants which is why it is marginally higher than the total eviQ webhits quoted previously. The latter includes data from medical, nursing, pharmacy, radiation therapy and unit webhits only. The top 100 pages visited accounted for 77% (n = 142, 537) of all page views. With respect to page content, 7 were registration/login pages, 45 were transition pages (such as tabs directing users to specific content) and the remaining 48 were cancer treatment pages [Table 2]. Registration/login and transition pages accounted for 85% (n = 122, 488) of the top 100 page views.

**Table 2:** Profile of the 100 most commonly accessed eviQ pages in October 2011

**Page type**	**n**	**Page views**	**Median time (range)**
General pages			
*Login/registration*	7	59,742	00:50 (00:26–01:53)
*Transition*	45	62,746	00:31 (00:11–01:46)
*Total*	52	122,488	00:31 (00:11–01:53)
Cancer treatment content pages			
*Protocols*	39	16,483	02:27(00:51–04:46)
*Supportive/Other*	9	3,566	02:23 (00:28–02:51)
*Total*	48	20,049	02:27(00:28–04:46)
**Total (top 100 pages)**	100	142,537	00:57 (00:11–04:46)

Users spent nearly five times longer on cancer treatment content pages (median 02:27, range 00:28–04:46) compared to registration/login and transition pages (median 00:31, range 00:11–01:53) [Table 2].

The most commonly accessed content pages (excluding login and transition pages) were medical oncology protocols (64/top 100 content pages); these were representative of the most common cancer types [Table 3]. Haematology (16/100) and supportive treatment (18/100) pages were also frequently accessed; supportive treatment pages included drug calculators, patient information, and side effect management pages (e.g., antiemetic regimens, extravasation management, neutropenia management).

**Table 3:** The 100 most commonly accessed cancer treatment content pages in October 2011

**Medical oncology (n = 64)**	**n**	**Median (range) page views/protocol**	**Total page views**
*Breast*	18	343 (138–1143)	7,770
*Colorectal*	19	238 (144–720)	5,879
*Respiratory*	11	163 (140–714)	2,783
*Gynaecological*	5	195(140–318)	1,014
*Upper Gastrointestinal*	5	235(158–515)	1,388
*Other solid tumor*	6	198 (156–279)	1,230
**Haematology (n = 16)**			
*Leukaemias*	4	177 (154–263)	770
*Lymphomas*	9	221(146–415)	2,155
*Myeloma*	3	241 (150–299)	690
**Radiation oncology (n = 2)**			
*Radiation oncology protocols*	2	145.5(140–151)	291
**Supportive treatment & other (n = 18)**			
*Drug calculators*	2	380(258–502)	760
*Management of side effects*	7	288(148–694)	2,108
*Patient information*	3	155(147–211)	513
*Other*	6	289 (145–432)	1,705

## Discussion

eviQ is an oncology protocol system that has been rated among the highest quality web-based oncology applications internationally [5,15]. This paper details a comprehensive examination of the uptake and patterns of eviQ use by healthcare professionals. Despite the increasing popularity of systems of this kind, this is the first study to explore the way in which clinicians are using web-based oncology resources.

Our study demonstrates eviQ is used by the key professional groups involved in oncology care. Currently, we are unable to ascertain the proportion of the oncology workforce registered with eviQ due to an absence of comprehensive data on the number of health care professionals employed in oncology practice in Australia. However, the distribution of health professional registrations by state is directly comparable to the proportion of cancer cases by state which suggests that eviQ has been adopted by clinicians nationwide [16]. This is promising in terms of the potential impact of eviQ on cancer treatment delivery across Australia.

The uptake of eviQ is not surprising given our previous research showing the system is perceived by Australian clinicians to provide high quality support for the full spectrum of cancer care [11]. The system also rates highly in terms of usability and applicability of protocols to clinical practice [13,14]. Our finding that the majority of registrants were referred to eviQ by their peers and colleagues is consistent with our previous work and demonstrates that the website is highly regarded by its users [11,12,17]. Our logfile analyses suggests that eviQ is used by clinicians at the point-of-care for cancer treatment delivery as most web activity (across all professional groups) occurred during standard clinic hours. Moreover, clinicians spent more time viewing specific chemotherapy protocols compared to other content and the cancer protocols represented the cancer burden for which chemotherapy is the standard of care (breast, colorectal, and respiratory cancers) [16,18]. Clearly, not all web traffic during standard clinic hours will relate directly to point-of-care treatment and our previous research has shown eviQ is used around the clock as a reference tool to prepare for clinics and research various treatment options [11,12].

Importantly, we found differences in the rates of eviQ use by health professional role and years of oncology experience. We found higher rates of use for oncology units compared with individual clinicians. This reflects real world clinical practice where numerous clinicians access the same computer and use a sole unit login during oncology clinics and the delivery of cancer treatment. Additionally, while pharmacists comprised one of the smallest individual clinician groups, they had the highest rates of use of any professional group. The more frequent use by pharmacists is likely due to their roles in the oncology treatment process: checking prescriptions/dose calculations and answering prescribing related questions on behalf of the multi-disciplinary team [11]. Additionally, clinicians with 5–10 years’ oncology experience (across all professional groups) had the highest rates of eviQ use (versus <5 and >10 years’ experience). It is highly likely clinicians with 5–10 years of experience are those working most actively at the point-of-care and making treatment decisions with less experienced staff working under their guidance. Further, our previous qualitative work demonstrated that the most senior oncology clinicians were the least reliant on eviQ to guide their practice [11].

In addition to better understanding the way in which different professional groups use eviQ our evaluation also highlights the utility of exploring use in multiple ways and the strengths and limitations of using specific indicators of use. To date crude webhits have been the standard eviQ metric. While this is a good indicator of the high level of web traffic, it tells little about the specifics of use. In contrast, rates are a valuable metric that allow for comparisons between different user groups. Our rate analyses could give the impression that individual level use is relatively low (an average of only 12 hits per month), however, one of the key limitations of our current analyses is the inability to examine the inter-registrant variability or to stratify analyses by frequent and non-frequent eviQ users.

The Google Analytics metrics (which account for extent of use) provide further detail on the content and time spent on eviQ during a typical session. However, logfile analyses are necessarily limited and should not be interpreted in isolation. Our program of work has made best use of other methodologies to address important questions relating to the use of computer support systems in oncology including system quality and clinicians perceptions about the utility of these systems [5,11,12,15].

## Conclusions

Our study has shown eviQ is used widely, registrations continue to grow and the system is becoming an integral part of Australian oncology practice. Globally, it is recognised that the number of cancer cases will increase, so too will the number of available treatments, placing higher demands on the oncology workforce delivering care [19]. As such, systems like eviQ are likely to play a more significant role in the safe and effective administration of cancer treatments. Ongoing monitoring and evaluations are pivotal to understanding the contribution of web-based decision support systems in promoting efficient service delivery, standardizing care and improving patient outcomes.

## Competing interests

The authors report no financial disclosures. RLW is the Program Director of eviQ Cancer Treatments Online and SR is a Cancer Institute NSW employee, which poses a potential conflict of interest- Cancer Institute staff may have a conflict of interest in evaluating their own website. The objective nature of the data and having external authors (SAP, JL, NP) responsible for the study design, data analysis and interpretation have reduced the potential impact of this conflict of interest.

## Authors’ contributions

JML contributed to design and conception of the study, data collection and analysis, interpretation and drafting of the manuscript. NP contributed to design and conception of the study, data collection and analysis, interpretation and drafting of the manuscript. SR contributed to data collection, interpretation and drafting of the manuscript. RLW contributed to interpretation and drafting of the manuscript. SP contributed to design and conception of the study, data analysis, interpretation and drafting of the manuscript. All authors read and approved the final manuscript.

## Pre-publication history

The pre-publication history for this paper can be accessed here:

http://www.biomedcentral.com/1471-2407/13/112/prepub
